# The SUMO pathway in pancreatic cancer: insights and inhibition

**DOI:** 10.1038/s41416-020-01119-6

**Published:** 2020-10-19

**Authors:** Christian Schneeweis, Zonera Hassan, Markus Schick, Ulrich Keller, Günter Schneider

**Affiliations:** 1 Medical Clinic and Polyclinic II, Klinikum rechts der Isar, Technical University Munich, 81675 München, Germany; 2 Department of Hematology, Oncology and Tumor Immunology, Campus Benjamin Franklin, Charité - Universitätsmedizin Berlin, Hindenburgdamm 30, 12203 Berlin, Germany; 3 German Cancer Research Center (DKFZ) and German Cancer Consortium (DKTK), 69120 Heidelberg, Germany; 4 Max-Delbrück-Center for Molecular Medicine, 13092 Berlin, Germany

**Keywords:** Targeted therapies, Targeted therapies

## Abstract

An urgent medical need to develop novel treatment strategies for patients with pancreatic ductal adenocarcinoma (PDAC) exists. However, despite various efforts in the histopathological and molecular subtyping of PDAC, novel targeted or specific therapies have not been established. Posttranslational modifications (PTMs) with ubiquitin-like proteins, including small ubiquitin-like modifiers (SUMOs), mediate numerous processes that can contribute to the fitness and survival of cancer cells. The contribution of SUMOylation to transcriptional control, DNA repair pathways, mitotic progression, and oncogenic signalling has been described. Here we review functions of the SUMO pathway in PDAC, with a special focus on its connection to an aggressive subtype of the disease characterised by high MYC activity, and discuss SUMOylation inhibitors under development for precise PDAC therapies.

## Background

Although the introduction of active chemotherapeutic regimens such as FOLFIRINOX and nab-paclitaxel with gemcitabine has considerably advanced the therapy of pancreatic ductal adenocarcinoma (PDAC),^1^ the 5-year survival rate of 9% for patients with this form of cancer remains unacceptably low.^2^ In addition to the substantial toxicities associated with these aggressive chemotherapeutic regimens, the low response rate in patients with metastatic disease underscores the need to develop new therapies.

PDAC heterogeneity, evident at numerous levels, is one substantial hurdle for the establishment of novel therapies. Two main subtypes of the disease have been consistently described: the basal-like subtype, which shows a strong overlap with the previously described squamous^3^ and quasi-mesenchymal subtypes,^4^ and the classical subtype.^5^ A 2020 study, which includes patients with advanced stages of the disease, further splits basal-like PDACs into basal-like A and basal-like B and the classical PDACs into a classical A and classical B type.^6^ Furthermore, a hybrid type of the disease showing expression of mRNAs belonging to identifier signatures of both main types has also been identfied.^6^ Classical subtypes of PDAC show an enrichment for stage I/II disease, whereas the basal-like A subtype is enriched in metastatic disease and seems to be resistant to current chemotherapies.^6^ The basal-like subtype is characterised by activation of the MYC pathway together with pro-inflammatory pathways, hypoxia networks, metabolic reprogramming, autophagy, epidermal growth factor and transforming growth factor-β (TGF-β) signalling and activation of the ΔNp63 pathway,^3^ as well as being enriched in mutations in the tumour-suppressor *TP53* and the lysine demethylase *KDM6A* and showing silencing of endodermal identity transcription factors, such as GATA6 or hepatocyte nuclear factor family members.^3,6–11^ The clear differences in the molecular underpinnings of the PDAC subtypes illustrate that understanding the biological mechanisms that drive these subtypes and connect the different subtypes with novel therapies represents one promising approach to improve the outcome of the disease.

**Fig. 1:**
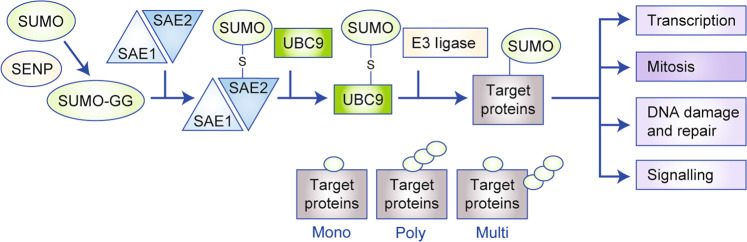
SUMOylation: a tightly balanced attachment of SUMOs to substrates. Small ubiquitin-like modifier (SUMO) proteins 1–3 undergo a maturation step, followed by an ATP-dependent SUMO transfer to the SUMO1-activating enzyme subunit 1 (SAE1)–SAE2 heterodimer and subsequent transfer to UBC9. UBC9 with contribution of E3 ligases transfers SUMOs to protein targets. The canonical functions of the pathway are depicted.

Posttranslational modifications (PTMs) are dynamic, reversible enzymatic modifications that regulate processes such as protein folding, cellular and subcellular localisation, activity, stability, and interacting partners. PTMs can ensure quick adaption to the continuously stressful extrinsic and intrinsic conditions faced by cancer cells, and accordingly, PTM with ubiquitin and ubiquitin-like molecules such as small ubiquitin-like modifier (SUMO) and neural precursor cell expressed developmentally downregulated (NEDD) can contribute to the fitness of cancer cells.^12–15^ Similar to the process of ubiquitination, the ~11 kDa SUMO protein is covalently conjugated to cellular proteins^19^ by an enzymatic cascade mediated by the concerted action of the E1 SUMO-activating enzyme (SAE), the E2-conjugating enzyme, UBC9, and a limited set of E3 SUMO ligases (Fig. 1 and Box 1).

Thousands of cellular proteins can be SUMOylated^18^ to ensure a highly dynamic regulation of cellular functions, such as protein localisation, stability, interactions, as well as the activity of targets. The protective role of the SUMO pathway for cells exposed to various cell-intrinsic, including alterations in the cellular redox state or DNA damage, and cell-extrinsic stresses,^19^ like hypoxia, is reflected by the upregulation of SUMOylation in cancer cells.^12^

SUMOs are predominantly found within the nucleus, and SUMOylation has thus been assumed—and in many instances proven—to critically modulate cellular processes associated with this compartment,^20^ ranging from the control of transcriptional processes, DNA repair, and mitotic progression to the regulation of cancer-relevant signalling pathways such as those mediated by TGF-β or nuclear factor-κB (NFκB). However, although a clear enrichment of SUMOylation events in the nuclear compartment is documented,^18^ cytosolic and membrane proteins can also become SUMOylated, which can further contribute to cancer-relevant functions of the pathway.^21^

The functions of the SUMO pathway in cancer have been reviewed elsewhere in detail,^12,13,20^ so we will focus in this article on the identified roles of the SUMO pathway in PDAC. We will describe the known functions of the SUMO pathway in PDAC and will summarise means and drugs to interfere with SUMOylation. We will explain how the SUMO pathway is connected to the MYC oncogene and how this relationship can be used to develop precise therapies by applying a concept of synthetic dosage lethality. Finally, we will describe future SUMO research directions to translate basic findings to the clinic.

Box 1: SUMOylation: a highly dynamic posttranslational protein modification Small ubiquitin-like modifier (SUMO)^17^ proteins 1–3 undergo a maturation step mediated by cleavage through sentrin-specific peptidases (SENPs)^16^ to generate the C-terminal diglycine (GG) motif. In addition, SENPs ensure reversibility of SUMOylation and contribute to recycle SUMOs. The most intensively studied SUMO proteins are SUMO1, SUMO2, and SUMO3, where the homology of SUMO2 and SUMO3 is 97%. In an ATP-dependent process, SUMOs are transferred to the SUMO1-activating enzyme subunit 1 (SAE1)–SAE2 heterodimer. SUMOs are bound via a thioester bond to SAE2/UBA2. Subsequently, SUMO is transferred to the indispensable E2-conjugating enzyme of the cascade, UBC9 (UBE2I), again forming a thioester bond. With contribution of E3 ligases (e.g. from the PIAS family), SUMO is transferred to the ε-amino group of lysine within protein targets via an iso-peptide bond. Protein targets can be mono-, poly-, or multi-SUMOylated. The canonical functions^12^ of the pathway are depicted in Fig. 1.

## The SUMO pathway in PDAC

The SUMO pathway has not yet been extensively analysed in PDAC. However, the importance of the SUMO pathway in other tumour entities, such as leukaemia or lymphoma, or many solid cancers, including breast, colorectal, or lung cancers^12^ together with investigations of the pathway in PDAC carried out over the past decade, underscores the potential relevance of this pathway with regard to the development of targeted therapies. Tissue-based analysis and mRNA expression profiles have defined an aggressive PDAC subtype that shows evidence of hyperactivity of the core SUMO pathway and thereby links the SUMO pathway with less-differentiated PDACs—the basal-like subtype—and an unfavourable prognosis.^22^ Mechanistic studies in PDAC link the SUMO pathway in particular with the response towards chemotherapies and to mechanisms of treatment resistance. Approximately 500 proteins were found to be modified by SUMO1 in untreated MiaPaCa2 human pancreatic cancer cells.^23^ Notably, treatment of PDAC cells with chemotherapy altered the SUMOylation state, and several target proteins became deSUMOylated or SUMOylated,^23^ underscoring the relevance of the pathway in cellular stress responses. Smad nuclear interacting protein 1 (SNIP1), for example, was found to be dynamically de- and re-SUMOylated in response to gemcitabine treatment;^23^ SNIP1 has a role in the survival of MiaPaC2 cells under acute therapeutic stress from gemcitabine treatment, which was found to depend on its SUMOylation.^23^

Perturbed SUMOylation equilibria are common in drug-resistant PDAC phenotypes. Promyelocytic leukaemia protein (PML) nuclear bodies are known to be involved in the regulation of cellular processes that are relevant to tumour suppression, such as DNA repair and the DNA damage response (DDR).^24^ The function of these nuclear organelles has been shown to be dependent on the appropriate SUMOylation of the major structural component PML, and hypoSUMOylation of PML in PDAC cells^25^ was associated with increased activation of the NFκB pathway to mediate gemcitabine resistance and increased activation of the cAMP response element-binding pathway to mediate oxaliplatin resistance.^25^ Importantly, a distinct heterogeneity of PML expression and PML SUMOylation was detected in patient-derived xenograft (PDX) models. A score integrating total PML expression and PML SUMOylation was positively correlated with patient survival. This observation was interpreted by the authors as indication for a decreased likelihood for responding to chemotherapy in the more aggressive, PML-score low population.^25^ To directly test PML as an indicator of chemotherapy responsiveness, PDX-derived cell lines were investigated. Indeed, higher levels of secreted PML, determined by proteomic analysis of secretomes, were connected with increased drug sensitivity.^25^ Although PML containing extracellular vesicles was described,^26^ the value of PML and its SUMOylation status in secretomes as a diagnostic marker for therapy responsiveness awaits further validation.

In addition, the E3-type SUMO ligase PIAS4 (protein inhibitor of activated STAT protein 4) was shown to be overexpressed in PDAC tumours and cell lines.^27^ Targeting PIAS4 by RNA interference reduced PDAC cell growth. Tumour cells are exposed to limited nutrient supply and hypoxia. Hypoxia is triggering adaptive signalling pathways to assure survival and rewiring of cellular metabolism, e.g. induction of glycolysis. The transcription factor hypoxia-inducible factor-1 (HIF1), composed of the hypoxia-regulated factor HIF1α and the constitutively expressed HIF1β, and the E3 ubiquitin ligase von Hippel–Lindau (VHL), which controls HIF1α protein abundance, are central regulators in this adaptive process.^28^ Under hypoxic conditions of PDAC cells, PIAS4 contributes to SUMOylation and inactivation of VHL.^27^ This molecular event is needed for the complete stabilisation of HIF1α in response to hypoxia. These data document the interaction of the SUMOylation- and hypoxia-triggered pathways in the context of PDAC, which further highlight the cross-signalling of both pathways at multiple levels.^12^

## Inhibitors of the SUMO pathway

**Fig. 2:**
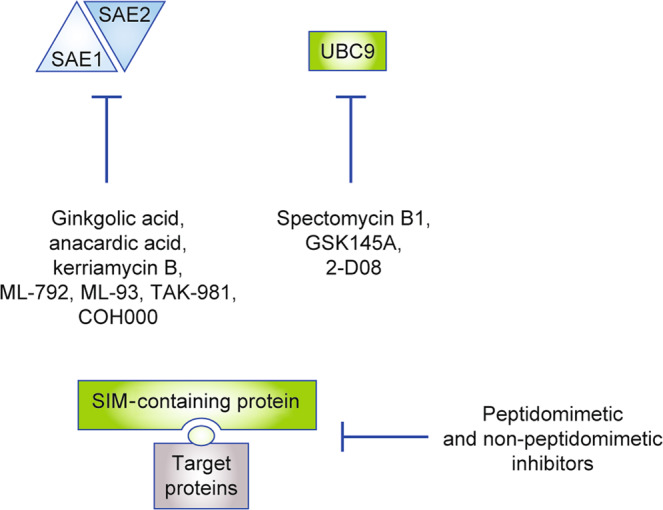
Inhibition of the SUMOylation pathway. The figure depicts inhibitors of the pathway, which block the SUMO-activating enzyme or UBC9. Furthermore, peptidomimetic or non-peptidomimetic inhibitors of the interaction of SUMOylated proteins with readers of the SUMO code using the SUMO-interacting motif (SIM) are shown.

Although the SUMO pathway has been implicated in cancer, only a few drugs targeting SUMOylation have been developed so far. Consistent with the existence of an enzymatic cascade to transfer SUMOs to its targets, the SUMOylation machinery can be targeted at several levels (Fig. 2) as well as at the level of SUMO maturation.

### Inhibitors of the E1 SUMO-activating enzyme

Several natural products have been described and used to target the SUMOylation pathway at the first activation step, which is executed by the E1 enzyme. Such compounds include ginkgolic acid and kerriamycin B, which have been shown to block the SAE complex (Fig. 2).^29^ Ginkgolic acid inhibits the growth of PDAC cell lines in the double-digit micromolar range in vitro and was also shown to be active in an in vivo xenograft model.^30^ However, mechanistically, ginkgolic acid was demonstrated to target a pathway driving lipogenesis; and any specific effects of ginkgolic acid on the SUMOylation machinery were not investigated in this study.^30^ The ginkgolic acid structural analogue anacardic acid inhibited the growth of PDAC cell lines in the double-digit nanomolar range. At the molecular level, the chromatin-modifying protein 1A was involved in the execution of the anacardic acid response and some activation of the ataxia telangiectasia mutated–p53 pathway was observed.^31^ Again, however, the role of the SUMOylation machinery was not investigated in this study, despite published evidence that anacardic acid reduces cellular overall SUMOylation.^32^ For example, in acute myeloid leukaemia (AML), anacardic acid reduced SUMO conjugation and increased cell death, especially in AML cells that were resistant to standard clinical chemotherapies.^33^

Due to the rather pleiotropic effects of naturally occurring SUMOylation inhibitors, synthetic and more specific inhibitors of the SAE complex have been developed. ML-792 and ML-93 form covalent adducts with SUMOs and thereby block SAE and the transfer of SUMOs to the E2 enzyme, UBC9.^22,34^ ML-792 and ML-93 in the nanomolar range reduce the fraction of SUMO-bound UBC9 and, consequently total protein SUMOylation, with no cross-reactivity with the NEDDylation and ubiquitinylation machineries;^22,34^ furthermore, when screened against 366 ATP-dependent enzymes, ML-792 demonstrated specificity for the SAE complex.^34^ ML-792 induces a failure of mitotic progression and chromosome segregation, with a consequent increase in endoreduplication and polyploidy.^34^ Both ML-792 and ML-93 inhibit the growth of PDAC cells and show a significant correlation in their half-maximal growth inhibitory concentrations in a large panel of murine PDAC cell lines.^22^ In sensitive pancreatic cancer models, ML-93 was effective in the double-digit nanomolar range, which was lower than the range observed using ML-792, resulting in the accumulation of cells in the G2/M phase of the cell cycle and in polyploidy with associated apoptosis.^22^ These results demonstrate the importance of SUMOylation for proper mitotic progression.^35^ An ML-792/ML-93-derived SAE inhibitor, TAK-981, entered clinical development in 2019, with Phase 1 trials recruiting patients with any advanced or metastatic solid tumour and lymphoma (NCT03648372 and NCT04074330).

In addition to SAE inhibitors that form adducts with SUMOs, a novel covalent inhibitor, COH000, which binds to Cys30 of SAE2, has been developed and characterised. COH000 does not compete with SUMO1 or ATP for binding to SAE but instead blocks the adenylation of SUMO^36^ by inducing conformational changes in the enzyme, demonstrating an allosteric mode of action.^37^ Functioning in the micromolar range in in vitro models, this inhibitor has demonstrated preclinical efficacy in colon cancer xenografts.^36^

### Inhibitors of the E2-conjugating enzyme

In addition to the E1 enzyme complex, the indispensable E2 enzyme of the SUMOylation cascade, UBC9, can be targeted.^29^ Genetically targeting UBC9 demonstrated the impact of this molecule as a fitness factor in PDAC.^22^ Although inhibitors of UBC9, which include spectomycin B1, GSK145A, and 2-D08^29^ have been described, no data for their efficacy in the context of PDAC are available.

### Potential SENP inhibitors?

As outlined earlier, the SUMOylation pathway is highly dynamic and reversible, and various SENPs ensure the deSUMOylation of target proteins and the recycling of SUMOs. Therefore, SENPs also represent candidates for pharmacological targeting, and synthetic inhibitors of these molecules are available.^29^ Despite conflicting data with respect to the overexpression of SENP1 in PDAC,^38,39^ triptolide, a natural product known to downregulate the expression of SENP1 in prostate cancer cells,^40^ acts in the double-digit nanomolar range in PDAC cell lines and has been shown to activate checkpoint kinase 2 (CHK2) in drug-sensitive lines, leading to inhibition of cellular growth.^41^ However, the direct involvement of SENP1 in this triptolide-induced cellular response is unclear. Furthermore, triptolide, which is also potent in selected in vivo PDX PDAC models,^42^ is known to target the ERCC3 helicase, a subunit of the transcription factor TFIIH^43^ to inhibit RNA polymerase II-dependent transcription. Therefore, the effects of triptolide on SENP1 seem rather indirect.

### Inhibiting SUMO-dependent protein interactions

Another means of interfering with the SUMO pathway is to prevent the recognition and interpretation of the SUMO code. The information imparted by SUMOylation is recognised and translated into altered biology by non-covalent interaction with other proteins that harbour a specific motif, the SUMO-interacting motif (SIM). Affimer (Adhiron) technology—a system based on artificial non-antibody scaffold proteins—was used to demonstrate that synthetic proteins blocking SUMO-dependent protein–protein interactions in a SUMO-isoprotein-specific fashion can be developed.^44^ For readers who are not familiar with this technology, please refer to refs. ^45,46^ Similarly, a SIM mimetic peptide coupled to gold nanoparticles interacts with poly-SUMO-2/3 chains and inhibits poly-SUMO-2/3-dependent protein–protein interactions.^47^ The involvement of the SUMO pathway in the control of the DDR could explain how the gold nanoparticle SUMO-2/3 ligand sensitises cancer cells to irradiation.^47^ Although non-peptidomimetic small-molecule SUMO–SIM inhibitors are under development,^48,49^ the therapeutic value in the context of PDAC is currently not known.

## MYC and its connection with the SUMO pathway

The basic helix–loop–helix leucine zipper transcription factor MYC dimerises with MAX, another basic helix–loop–helix leucine zipper transcription factor, to bind to enhancer (E-) boxes in the promoters of numerous genes. As an oncoprotein, MYC controls the metabolism, growth, and proliferation of cancer cells.^50^ Witkiewicz and colleagues showed that amplification of *MYC*, which was found in around 14% of patients with PDAC in their study, is the sole copy number variation associated with the poor survival of PDAC patients.^51^ Furthermore, another study showed that *MYC* amplifications occur more frequently in liver metastasis (12%) than in primary pancreatic tumours (4%) and lung metastasis (6%), highlighting an important function of MYC along the route to liver metastasis.^52^ This observation is underscored by the demonstration that amplifications of *MYC* are positively selected for during tumour progression,^53^ as well as the finding that MYC activity is associated with the basal-like subtype of the disease.^3,6^ Therefore, targeting MYC and MYC-dependent pathways could offer opportunities for novel therapies for patients with advanced disease and very poor prognosis who might be resistant to currently established standard therapies. Potential approaches to target MYC in the context of PDAC, which include bromodomain and extra-terminal motif (BET) inhibitors or MYC-MAX dimerisation inhibitors, have been described previously,^54–56^ so we will focus here on the concept of MYC-associated synthetic lethality.^57^

### Synthetic lethality

Synthetic lethality usually refers to the situation in which individually targeting each gene within a pair of genes is tolerated but the combined inactivation induces a dramatic loss of cancer cell fitness. Synthetic lethality can also occur between genes and small molecules, as exemplified by the sensitivity of tumour cells harbouring mutations in the DNA repair gene *BRCA1/2* to poly-ADP ribose polymerase (PARP) inhibitors and certain chemotherapies, such as platinum compounds.^58,59^ As the genetic lesion is restricted to cancer cells, therapeutic concepts based on synthetic lethality might open an exploitable therapeutic window. Indeed, the benefits of platinum therapy or PARP inhibition have been demonstrated in patients with *BRCA1/2*-mutated PDAC^60–62^ supporting the notion that synthetic lethality is relevant in PDAC and that there is a need for preclinical and clinical research to improve understanding of such concepts.

### MYC and synthetic dosage lethal interaction

Notably, a specific kind of synthetic lethality, called synthetic dosage lethality, defines a situation in which hyperactivity of one gene generates a dependency on another gene product, and is relevant in the context of the MYC pathway. Similar to the situation for genetic lesions, the restriction of the hyperactivation to cancer cells provides an exploitable therapeutic opportunity. Accordingly, several unbiased genetic screens have validated the concept of synthetic lethality associated with the MYC protein family,^57,63–70^ and these screens have been supported by many observations that postulate a synthetic lethal relationship of MYC with the splicing machinery and the arginine methyltransferase PRMT5,^71^ CHK1,^72^ cyclin-dependent kinase 1/2,^73–75^ Aurora kinases,^76–78^ death-receptor engagement,^79^ PIM1,^80,81^ BET inhibition,^82,83^ polo-like kinase 1,^84^ the mitotic machinery,^85,86^ and protein homoeostasis.^87,88^ These data suggest that MYC drives the cellular machineries that are responsible for splicing, protein homoeostasis, transcription, replication, or mitosis, to a limit beyond which cells cannot cope with any additional stress targeting these particular processes. Therefore, MYC marks cancers with a specific set of therapeutic vulnerabilities, which should consequently facilitate the stratification of patients for precise therapeutic interventions.^56^

### The MYC–SUMO connection

**Fig. 3:**
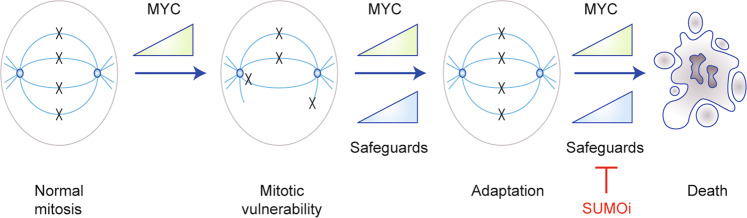
MYC and SUMO inhibitor sensitivity. The association of high MYC activity to the sensitivity of PDAC cells to SUMO inhibitors is depicted. Increased expression of MYC leads to mitotic alterations and generates vulnerabilities. Therefore, cancer cells with high MYC activity depend on safeguard pathways to cope with this particular stress. These safeguard pathways, which include the SUMOylation machinery, allow the cells to adapt to mitotic stress. Blocking the SUMO pathway induces G2/M phase cell cycle arrest, polyploidy, and subsequent cell death. Beyond the SUMO pathway, mitotic genes, like TPX2, BIRC5/survivin, and EG5/kinesin‐5, can function as safeguards. Please see also the two recent publication of the Goga^93^ and Taylor^92^ laboratories for a detailed discussion.

In 2012, an unbiased genetic screen demonstrated the synthetic lethality of the SUMO pathway components SAE1 and SAE2 with MYC,^64^ an observation subsequently corroborated in haematological malignancies^89^ and small cell lung cancer (SCLC).^90^ Across species, MYC-driven B cell lymphomas were characterised to upregulate core components of the SUMOylation machinery, including SUMO proteins, SAE1, SAE2, and UBC9. Inhibition of the SUMO pathway triggered a G2/M phase arrest of the cell cycle, polyploidy, and apoptosis in a MYC-specific manner. Genetic interference with the pathway by targeting SAE2 demonstrated therapeutic efficacy in murine and human B cell lymphoma models in vivo.^89^ Furthermore, the knockdown of SAE2 in SCLC cell lines with high MYC expression induced an increased therapeutic effect compared to lines with low expression of the oncogene.^90^ Work investigating the SUMO pathway in context of MYC showed that the SUMOylation pathway is required to cope with MYC-induced mitotic stress^64^ and that MYC hyperactivation in the context of SUMO inhibition results in irregular spindle activity, aneuploidy, and subsequent apoptosis.^64^ The concept is illustrated in Fig. 3.

In contrast to the well-known functions of MYC in the G1 or S phases of the cell cycle,^91^ the role of MYC in mitosis is less well understood. Cells with high MYC levels show morphologically altered spindles and are characterised by changes in the timing of mitotic progression.^92^ Furthermore, increased MYC expression is associated with misaligned chromosomes in metaphase with subsequent lagging chromosomes in anaphase.^93^ This association is relevant from the therapeutic view, as MYC was demonstrated to be a critical determinant of cell fates occurring upon the treatment of cells with various perturbations affecting mitosis.^86,92^ Such observations are clinically relevant, as demonstrated by the increased responsiveness of *MYC*-amplified breast cancers to docetaxel-containing neoadjuvant chemotherapies.^94^ Considering that the SAE inhibitor ML-792 interferes with mitotic progression and chromosome segregation,^34^ the increased potency of such SAE inhibitors in *MYC*-hyperactive solid cancers seems well explained.

At the molecular level, a 2020 study implicates the microtubule-binding protein targeting protein for *Xenopus* kinesin-like protein 2 (TPX2) as an *MYC*-associated synthetic lethal gene.^93^ TPX2 is required for spindle assembly during mitosis and the gene is directly activated by MYC. Whereas normal cells need low amounts of the protein for spindle assembly, cancer cell with high *MYC* expression depend on TPX2 to efficiently form the spindle and progress though mitosis.^93^ Importantly, the synthetic lethal interaction of MYC is not restricted to TPX2. Synthetic lethality between MYC hyperactivation and the mitosis regulators BIRC5/survivin and EG5/kinesin‐5 was also demonstrated,^93^ and MYC-associated synthetic lethal screens were enriched for mitotic genes.^64^ Interestingly, many proteins identified in large-scale proteomic screens to harbour multiple SUMO sites are mitotic proteins, and BIRC5, EG5, and TPX2 can all be SUMOylated.^95^ In fact, 39 SUMO sites have been identified in TPX2.^96^ This high number could function to ensure proper spindle formation by stabilising interactions with other spindle factors. As SUMOylation plays an important role in the organisation of the spindle and kinetochore,^95^ it will be interesting to determine whether altered SUMOylation of these mitotic proteins is involved in the response of the cell to SUMO pathway inhibitors.

Importantly, the connection of MYC to the SUMO pathway is relevant in PDAC. PDAC cells with higher *MYC* expression tend to exhibit increased overall SUMO1 and SUMO2/3 protein SUMOylation and increased expression of core SUMO pathway genes.^22^ In large panels of human and murine PDAC cell lines, ML-93 sensitivity correlated with MYC hyperactivity, and growth inhibition with ML-93 monotherapy was observed in a xenograft model.^22^ In dynamic re-population assays, the selection pressure of ML-93 treatment conferred a definite growth disadvantage on the MYC hyperactive population, and the association of MYC hyperactivity with an increased sensitivity to SAE inhibitors was confirmed in conditional ‘MYC-on’ models, which depend on a tamoxifen-activatable MYC oestrogen-receptor fusion protein.^22^ Again, also in the context of PDAC, the SAE inhibitor induced an accumulation of cells in the G2/M phase of the cell cycle, providing evidence that this particular targeted therapy is triggering a mitotic vulnerability.

## Future directions

First evidence implicates that SUMO PTM represents a dynamic biomarker for the response towards currently used chemotherapies.^25^ These findings offer the opportunity to validate the expression of PML, for example, and to measure its SUMOylation status in prospective clinical trials to select for chemotherapy responders. The data demonstrating that various cellular stresses induce dynamic SUMOylation and deSUMOylation events argue for systematically studying SUMOylation targets as well as the processes that are controlled by these events. This approach will provide information to develop novel molecular-informed and rational mechanism-based therapies. In addition, investigating the specific role of SENPs, which, in normal cells, tightly control the SUMOylation equilibrium, could provide information for additional pharmacological intervention in MYC/SUMO-activated PDAC and other cancers.

The development of SAE inhibitors clearly shows that specific inhibition of the SUMO pathway is feasible. Despite the critical importance of SUMOylation, SUMO inhibitors globally targeting SAE are well tolerated in preclinical models^22^ and Phase 1 clinical trials are currently ongoing. The investigation of such inhibitors in the clinic (e.g. TAK-981) and the development of novel highly specific SUMO inhibitors will allow for testing the principle of SUMO inhibition in molecularly informed translational applications. The efficacy of clinical SUMO inhibitors will, however, depend on the development of stratification concepts for selecting PDAC patients with tumours that are particularly sensitive to this approach.

Current data argue that SUMO inhibition is relevant for MYC-hyperactivated PDACs. However, not all PDAC models with evidence for MYC hyperactivation respond to the SAE inhibitors.^22^ This lack of response towards targeted therapies despite the selection by a molecular marker is typically seen in a portion of patients with gastrointestinal cancers in the clinic.^97^ However, biomarker-driven therapy selection has been successful in other cases.^97,98^ Therefore, to proceed with the concept of SUMO inhibitors for PDAC, several additional issues must be addressed. First, the response of PDAC cells to SUMO inhibitors must be analysed in greater functional detail, which might additionally allow to characterise the MYC-hyperactivated cancers with very high sensitivity towards SUMO inhibition. Furthermore, other markers in addition to MYC should be considered to define the SAE inhibitor-sensitive proportion of PDACs more precisely. Multivariate models have been shown to predict responses towards immunotherapies with high accuracy.^99^ Considering that the full oncogenic power of MYC is modulated by several co-factors,^100^ a similar approach might point to a path for defining multivariate predictive models. Clinical data from the past 5 years implicate the value of combining two or more targeted therapies to treat solid cancers as exemplified for colon cancer.^98^ Therefore, SUMO inhibitor combination therapies could be developed in order to treat MYC-hyperactivated PDACs. The demonstration that ML-792 does not synergise with chemotherapies that act in the mitotic phase, including paclitaxel,^34^ argues for the need to systematically screen for such combination therapies. In addition, the first-in-class SUMO inhibitor, TAK-981, demonstrated immune-modulating properties.^101,102^ Considering that MYC mediates complex cross-talk between tumour cells and the tumour microenvironment,^103,104^ SUMO inhibitors should also be tested in autochthonous immune-proficient models.

Current knowledge about the SUMO pathway and the response to SUMO inhibitors in the context of PDAC will not immediately enable SUMO pathway targeting therapies to be applied in the clinic. However, the importance of the pathway, the existence of specific inhibitors, and evolving concepts of synthetic lethality should enable the development of such novel therapies for PDAC and should be advanced for an aggressive subtype of the disease that is largely resistant to current standard therapies.

## Data Availability

Not applicable.
